# Transforming the practice of medicine through team science

**DOI:** 10.1186/s12961-020-00619-4

**Published:** 2020-09-17

**Authors:** Jason H. Pitzen, Heidi L. Dieter, Darren L. Gronseth, Amber K. Dahl, Venessa L. Boyle, Tharana Maran, C. Michel Harper, Gregory J. Gores

**Affiliations:** 1 Department of Planning Services, Mayo Clinic College of Medicine and Science, Mayo Clinic, Rochester, MN United States of America; 2 Department of Research Administration, Mayo Clinic, Rochester, MN United States of America; 3 Department of Management Engineering & Consulting, Mayo Clinic, Jacksonville, FL United States of America; 4 Department of Neurology, Mayo Clinic College of Medicine and Science, Mayo Clinic, Rochester, MN United States of America; 5 Division of Gastroenterology and Hepatology and Department of Physiology and Biomedical Engineering, Mayo Clinic College of Medicine and Science, Mayo Clinic, 200 First St SW, Rochester, MN 55905 United States of America

**Keywords:** Diffusion of innovation, Organisational innovation, Translational medical research

## Abstract

**Background:**

The translation of biomedical research discoveries into clinical practice is marked by extended timelines (averaging 17 years) and multiple sequential process steps. However, even after a drug, device, diagnostic tool or unique therapeutic procedure successfully navigates through clinical testing to approval, real barriers remain in applying and scaling the innovation in practice.

**Methods:**

Mayo Clinic initiated the Transform the Practice programme to facilitate multidisciplinary team and convergence science to continuously reinvent solutions to address unmet patient needs and accelerate the application of next-generation healthcare solutions. During a 5-year period, 24 programme teams received financial resources, barrier-removing engagement from clinical and research leadership, and enhanced administrative support, including dedicated project managers.

**Results:**

The approach created value in facilitating consistent progress toward project objectives and resulted in multiple publications, new extramural funding sources, and implementation of new tests and services into the clinical practice. This report describes the concentrated institutional effort to accelerate the discovery–translation–application continuum in an academic medical centre and highlights successful applications and persistent obstacles.

**Conclusions:**

The Transform the Practice approach is effective in moving high-potential research discoveries closer to implementation in the clinical practice. Its concepts, including the application of structured project management methodology, may be quickly integrated to shorten an organisation’s time to implementing its most important discoveries.

## Background

Investment in medical and health research and development in the United States reached $171.8 billion in 2016 [1], roughly 1% of the United States gross domestic product [2]. Although this sum corresponds to more than 45% of research and development spending by businesses in all industries [3], the average lag time from a biomedical research discovery to its translation into the clinical practice is 17 years [4, 5]. Hanney et al. outline the challenging nature of measuring this lag time and assert that the duration is likely even longer if standardised time points from basic research to health benefits are employed [6].

Such a prolonged time to market, which would be unfathomable in other industries, represents lost opportunities to bring treatments and diagnostic tools to patients as well as a loss of economic value. A United Kingdom study, for example, estimated that cardiovascular disease research had a 9% rate of return on investment that was attributable to a direct health improvement, assuming the 17-year lag. Reducing the lag to a still-unimpressive 10 years increased the rate of return by 4 percentage points (to 13%) [5]. Similar returns have subsequently been estimated for musculoskeletal disease research (7%) and cancer research (10%) [7, 8].

Perhaps the time to translate a biomedical research discovery is unsurprising, given the number of steps between the laboratory and bedside that are intended to protect patients from unintended harm and to ensure efficacy. Such steps are usually required by regulatory bodies; in the United States primarily by the Food and Drug Administration. For a new pharmaceutical agent, these steps include early discovery, target identification, lead optimisation, preclinical testing, investigational new drug application, multiphase clinical testing (with phases I, II, III and often IV) and, finally, a new drug application [9].

In 2011, the National Institutes of Health (NIH) Director Francis Collins announced the establishment of the National Center for Advancing Translational Sciences. He highlighted the need to improve the development pathway for diagnostics, devices and therapeutics, referencing the “*triple frustrations*” of long times to translation, high costs and high failure rates [10]. Collins asserted that biomedical research showed strong technological progress at the basic science level, which was traditionally heavily funded by NIH, with subsequent clinical trials that were funded by private companies with financial incentives to move promising agents into the clinical practice. The problem area, according to Collins, was the middle of the pipeline, with countless potential barriers between laboratory research and late-phase clinical studies. Complicating the matter, as Trochim et al. point out, are the difficulties inherent in evaluating a translational process that rarely proceeds in an organised, step-wise manner [11].

However, the true problem extends even further, with considerable hurdles slowing the translation of a discovery to a clinical study as well as its application into clinical practice. Morain [12] highlighted this problem with the poignant example of Zika virus, which went from a relatively unknown virus before 2015 to becoming the subject of at least 3000 papers by 2017. Although this research led to the Centers for Disease Control and Prevention recommending imaging and testing for infants exposed to Zika, adoption of these recommendations was painfully limited in the care setting. Only a quarter of infants exposed to the virus underwent imaging and less than two-thirds were tested for Zika infection.

The slow adoption of Zika guidelines illustrates the widespread failure to meet the modern expectation that research produces knowledge that can be applied to meet a market need, rather than producing knowledge purely under the auspice of societal good [13]. Of all the actions required to bring novel healthcare solutions to patients, the process from discovery through translation is only the beginning, and it may be the easy part. The subsequent and more elusive component is application, which involves spreading the word about the innovation and getting payers to pay, providers to order and patients to consent. In fact, the dissemination and implementation of evidenced-based interventions has become a science of its own [14].

Traditionally, innovations are shared with professionals through academic publications; however, approximately 28,000 peer-reviewed English-language scientific journals are publishing about 2.5 million articles annually [15]. Advances in artificial intelligence may someday increase our consumption of information but, currently, we cannot expect payers, providers and patient consumers to seamlessly filter through and select from a menu of innovations. A proactive approach is necessary to intentionally manage discoveries through translation and application in the clinical setting.

In 2014, we began a concerted approach to address these obstacles at the Mayo Clinic. Many obstacles to applying discoveries to medical care are external to the institution, as noted above, but even within a single academic medical centre, significant barriers exist. For example, multidisciplinary team and convergence science requires intentional alignment of incentives management and resources to be successful. Within an academic medical centre, the department model and time constraints created by clinical practice priorities hinder the acceleration of the discovery–translation–application (DTA) process. After a refresh of the Mayo Clinic strategic and operating plans and identification of several vital initiatives, the executive deans for practice and research partnered in 2014 to launch Mayo’s Transform the Practice (TtP) initiative. The initiative invited multidisciplinary teams of physicians and scientists from across the DTA continuum to apply for support and funding that would fast-track the implementation of new therapeutic agents or diagnostic tools into practice, with the overriding goal of addressing the unmet needs of our patients. During the past 5 years, 24 teams participated in the programme. Herein, we review the successes and failures of this initiative.

## Methods

The executive deans for practice and research assembled the TtP goal team to address Mayo Clinic’s strategic goal to transform the practice of medicine. In addition to the two deans, the team included the chair and vice chair of research administration, the institution’s chief planning officer, a research administrator and a planning administrator. Collectively, the TtP goal team was responsible for executing three strategic objectives, as follows: (1) facilitate multidisciplinary team science; (2) continuously reinvent and differentiate care for serious and complex diseases; and (3) accelerate the application of next-generation healthcare solutions.

To begin, the TtP goal team developed a process for conducting an annual request for applications (RFA). Teams were invited to respond to the RFA by using an abbreviated application (Box 1). Prospective teams were provided with numerous evaluation criteria. The RFAs placed no upper or lower limits on the dollar amount that could be requested and resource requests could span the entire spectrum of needs supporting translational research projects. Teams could request up to 2 years of support.

**Fig. 1:**
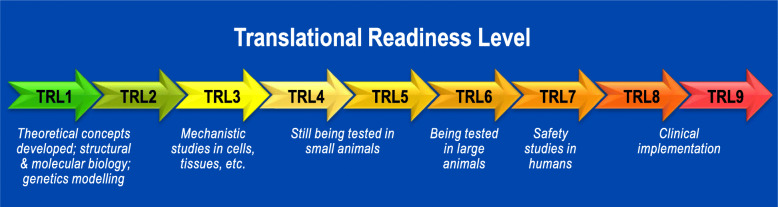
Translational readiness level

Applicants assessed the translational readiness level (TRL) of their innovation (Fig. 1). The TRL for medical research was adapted from the United States Department of Defense Technology Readiness Assessment Deskbook of 2009 [16]. The TRL includes representative hurdles from discovery to translation and was used to measure the distance between an innovation and its implementation into the clinical practice. After 2 years of using the TRL, it became clear that teams were overestimating how far along they were on the TRL scale. To clarify applicant expectations, the RFA criteria were revised to indicate that the research project was required to be ready for application in the practice within 2 years.

Applications were subjected to an NIH-style review process and select applications underwent detailed review by established physicians and scientists within the organisation. This panel assessed the scientific merits of the applications, their alignment with institutional plans and priorities, and the expected time to implementation. We identified additional stakeholders and resources for projects aligned with institutional priorities to ensure the success of the projects. In addition, the vice chair of Mayo Clinic Ventures, the organisation’s technology transfer office, participated in assessing the innovation’s commercial potential because the TtP goal team recognised that commercialisation was an efficient pathway to translation and application. Department chair support was critical and required for project approval. The TtP goal team had previously observed that, when department chairs were invested in a project, barriers to implementation were reduced. After the final teams were identified, the executive dean for research and vice chair for research administration met with department chairs to identify the support that they would provide to ensure the success of the projects.

After project teams were notified, the TtP goal team assigned project manager resources. The amount of full-time equivalent project manager time assigned for each project varied, but the most common allocation was approximately 10%. The project manager was introduced to the team at a project kick-off meeting. The project manager’s first task was to work with the team to translate the scientific proposal into a project charter, which described the project’s purpose, key milestones, time frames and resource requirements. The charter was then entered into Mayo Clinic’s system for project management, tracking and reporting. In many cases, other tools were used to manage the more complex elements of the project.

Project managers assisted their teams by removing barriers that were delaying or preventing the translation process. To accomplish this, they acted as liaisons to departmental administrators, the research administration vice chair, and the TtP goal team (if needed) and recommended actions to remove barriers that the teams were encountering. As the projects progressed, project managers communicated to internal business units that the projects were institutional priorities and should be treated accordingly. A key responsibility of the project managers was the quarterly status report. These reports described the team’s progress, identified barriers, risks and the actions being taken to overcome them, and conveyed the overall status of the scope, schedule and budget for the project. Status reports were reviewed by the TtP goal team to identify any actions needed to help the team overcome the barriers it identified.

From 2014 to 2018, the TtP initiative selected 24 teams representing diverse specialties (Box 2). In 2017, health systems engineers from Mayo’s internal business consulting group, Management Engineering & Consulting (ME&C), partnered with TtP to provide an operational assessment and gather feedback from the TtP teams. ME&C engineers interviewed principal investigators (PIs) and project managers from 18 TtP teams (established from 2014 to 2017) to perform an objective analysis from a systems thinking perspective. Points of feedback were categorised as Strengths, Weaknesses, Opportunities or Threats (SWOT).

In June 2018, the TtP research administrator emailed questionnaires to the PIs of the first 18 teams that were established from 2014 through 2017. Surveys included the following questions: (1) Did the project result in a new treatment, test or diagnostic tool? (2) Did the project result in additional extramural funding? (3) Were the results published? (4) Has there been any outside commercialisation or licensing? Respondents were asked to provide details for any affirmative answer.

Box 1. Summary of the Transform the Practice request for applications Components of the application

-  Team members
-  Project goals
-  Preliminary data
-  Specific aims
-  Research plan
-  Expected outcomes
-  Use of existing assets and resources
-  Alignment with goals of the Transform the Practice initiative
-  Translational readiness level
-  Resource needs

Project evaluation criteria

-  Aligns with clinical priorities
-  Shows preexisting engagement with clinical priority areas
-  Creates new diagnostic tools or therapeutic advances
-  Addresses unmet clinical needs
-  Helps generate patient demand
-  Includes a multidisciplinary team of physicians and scientists with skills relevant to the discovery-translation-application research continuum
-  Extends organization-wide or can easily be scaled across geographic sites
-  Leverages existing resources, infrastructure, and assets
-  Includes department chair support

Research requests

-  Coordinators (study, data, nurse)
-  Laboratory technologists
-  Project managers
-  Statisticians
-  Postdoctoral fellows
-  Device or cell manufacturing costs
-  Patient care (procedures, laboratory tests, imaging)
-  Supplies and equipment
-  Facility fees
-  Pharmacy
-  Regulatory costs (e.g. FDA approval)
-  Core facility services

**Abbreviation:** *FDA* US Food and Drug Administration

Box 2. Transform the Practice team projects, 2014–2018

- Biomarker for Adrenal Masses
- Continent Ileostomy Valve
- Early Detection of Endometrial Cancer by Cellular DNA
- Focal Cartilage Repair in the Knee
- Gastrointestinal Fistula Repair
- Genetic Assessment of Pediatric Very Early Onset Inflammatory Bowel Disease
- Genetic Stratification of Glioma Patients
- Immunotherapy for Lymphoma
- Immunotherapy Using Cryoablation and Intratumor Dendritic Vaccine
- Larynx Regeneration by 3D Biomatrix Printing
- Lymphedema Prevention After Breast Surgery
- Melanoma Therapy
- Mesenchymal Stem Cells for Treatment of Degenerative Disc Disease
- Molecular Breast Imaging
- Molecular Prognostication of Prostate Cancer
- Noninvasive Assessment of Pediatric Cardiac Allograft Vasculopathy
- Noninvasive Screening and Detection of Bladder Cancer by Proteomics
- Oncolytic Virus Therapy for Pediatric Brain Tumors
- Osteoarthritis Gene Therapy
- Proteasome Inhibitor Therapy for HIV
- Replacing Liver Biopsy with Magnetic Resonance Elastography
- Stem Cells for Coronary Microvascular Dysfunction
- Treatment of Spinal Cord Injury by Neuromodulation
- Treatment of Traumatic Spinal Cord Injury by Mesenchymal Stem Cells

## Results

The ME&C analysis indicated that the main factors slowing the progress of the TtP teams were institutional barriers, absent or ambiguous resources, delays in patient recruitment, and lack of clarity about the project manager’s role. A detailed examination of barriers highlighted differences in team goals. For example, teams working to design and develop products or diagnostic tools encountered progress delays that differed from those of teams conducting clinical trials.

**Fig. 2:**
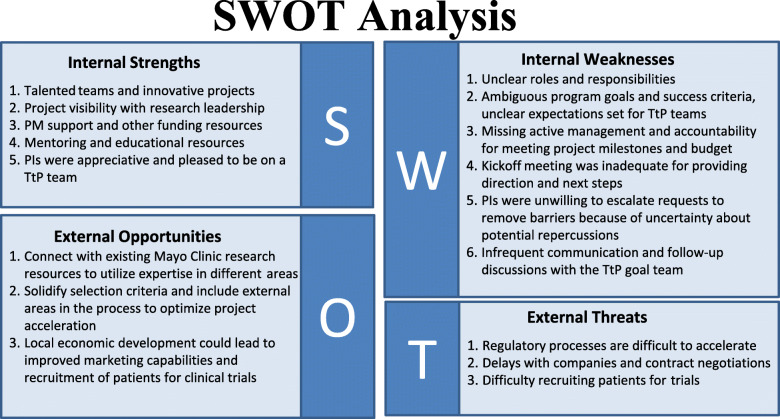
Strengths, Weaknesses, Opportunities or Threats analysis. *PI* principal investigator, *PM* project manager, *TtP* Transform the Practice

The SWOT analysis outlines TtP’s competitive position within the Mayo Clinic (Fig. 2). Interviews showed strong appreciation for the opportunity provided by the TtP initiative, including resources and access to project manager support. Interview comments also indicated early challenges with incorporating the project management discipline into biomedical translation projects as well as a lack of clarity about the criteria for success and accountability within the TtP framework. External delays associated with regulatory processes, contract negotiations and patient recruitment to clinical trials also affected TtP projects.

In response to the feedback obtained by ME&C, TtP implemented improved procedures for 2018 teams. At project kick-off meetings, the roles and expectations of each participant (e.g. PI, project manager, administrator and coordinator) were outlined. In addition, the escalation path for issues was defined; it started with engaging the PI’s existing clinical or research operations manager, followed by the vice chair of administration and then the TtP goal team (if needed). For better budget tracking, ongoing review of the project’s financial position was added to the PI’s regularly scheduled programme financial review meetings. Long-term improvements were identified, including collaborating and building relationships with other departments in the organisation, customising the project manager role and developing a programme model that could be followed by the project team.

Some projects had timelines to clinical application that were slower than anticipated. As causes for delays were explored, we developed additional selection criteria for future teams. We asked the following questions: (1) Is participant recruitment feasible? (2) Is the PI an established investigator with infrastructure to support the project or a new investigator who needs time to build a team? (3) Does the project pertain to test development and, if so, is it a priority for laboratory medicine? (4) Is the protocol developed and ready for review by the institutional review board? (5) Is a 2-year time frame realistic to attain goals? (6) Do external companies need to be involved in tasks (e.g. provision of a drug) that will increase time to implementation?

In 2018, 17 (94%) responses were received to a survey of the first 18 TtP teams, including one from a PI who requested an in-person interview (this respondent’s data are included in the results summarised below). Five (29%) respondents indicated that their project resulted in a new treatment, test or diagnostic tool. Seven (41%) indicated that they had secured new extramural funding through benefactors, foundations or government-sponsored grants. Six (35%) indicated that manuscripts reporting project findings were published, accepted for publication or were under review for publication. None of the respondents indicated outside commercialisation or licensing at the time of the survey.

**Fig. 3:**
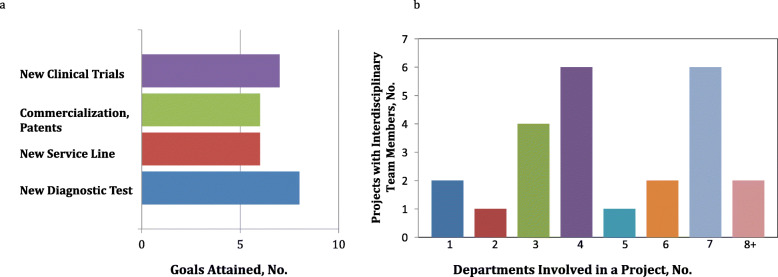
**a** Outcomes of Transform the Practice team projects. Outcomes were assessed in 2018 and 2019; teams were formed from 2014 through 2018. **b** Interdisciplinary teams supported by Transform the Practice

In the summer of 2019, we contacted the 24 teams that were selected from 2014 through 2018 to review any new successes of their TtP projects (Fig. 3a). In total, 18 (75%) teams had achieved at least one favourable outcome. Seven (29%) teams reported a new or subsequent phase clinical trial, 6 (25%) teams had advanced a product toward the commercial market or obtained a patent, 6 (25%) teams had implemented a new clinical service line, and 8 (33%) teams had developed a new diagnostic test. We also observed that most teams (21/24, 88%) included members from three or more departments (Fig. 3b).

## Discussion

Academic centres have not consistently advanced from collaborations to a mature and harmonious level of team science, i.e. teamwork across disciplines and specialties. Initial success in the academic research environment is still often represented by the achievement of an independent research laboratory with substantial extramural funding. Successful translational research requires a multidisciplinary team science approach, with leaders who recognise the benefits of a diverse team and are able and willing to invest in the infrastructure to support these teams [17]. The TtP initiative exemplifies Mayo’s model of research, which focuses on the entire DTA continuum through the underpinnings of team science. Further, TtP has fostered a truly interdisciplinary approach, as evidenced by the majority of teams having members from at least three departments.

The concepts underlying TtP are necessary to continue advancing the practice of medicine. TtP supports talented study teams and unique ideas that have the capability of transforming the care delivered to patients. These teams are provided visibility across the organisation, funding, mentoring and educational resources. In return, national programmes are being developed to address the unmet needs of the patients and enhance clinical demand by accelerating research into practice solutions. Project managers bring rigor to the planning, organisation and progress toward milestones. Although project management still is not widely accepted in academia, it has a promising role as a linchpin that supports team science and the overall goals of accelerating translation and application [18].

The TtP goal team was a small group of institutional leaders who were able to quickly remove barriers as they were identified, which allowed the teams to continue moving forward. Resources were also provided that consisted of monies for supplies and reagents, research coordinators, project managers, writing-off clinical charges, and partial full-time equivalent support. The amount varied markedly between projects and is difficult to extrapolate between institutions given the varied accounting practices and cost structures. Once resource allocation was determined, having one central person as a point of contact was helpful when addressing system-wide questions and issues, instead of addressing them one team at a time. Having the executive dean for practice and the executive dean for research involved in selecting and backing the teams led to a common recognition of the projects that should be prioritised across the institution and effectively ensured that the research focus was aligned with practice priorities and unmet patient needs.

Indeed, the simple concept of institutional (private and public) prioritisation of research projects is playing out before our eyes during the COVID-19 pandemic. As Hanney et al. describe in follow-up to their previous work, the attention and prioritisation provided at every level for COVID-19 vaccine development will most likely lead to health benefits in a small fraction of the 10 years usually experienced for vaccines [19]. TtP at a local institutional level and COVID-19 at a worldwide level offer strong evidence for greatly improved time to application when priorities are aligned and resources are mobilised.

Although institutional leadership prioritised the TtP-supported research projects, administrative barriers were still encountered at the local level. Historically, administrators supporting clinical departments and divisions were not closely involved in research projects because they relied heavily on their research administrator colleagues. However, because the ultimate goal of each selected team was to move their research project from translation to application into a clinical department or division, engagement of the clinical administrator was critical from the very beginning. This early engagement helped remove barriers in the practice.

Considering the lag time from biomedical research discovery to application into the clinical practice, the 2 years of support from the TtP initiative was insufficient for some research teams and projects. This insufficiency is further exacerbated for teams still in the discovery phase and for teams applying for assistance to bridge the ‘valley of death’ between bench science and translation. The different TtP teams highlighted variations in the process and speed that a project could progress at toward clinical application. The meaningful progress in the year between surveys solidified the need to continue to track clinical practice application and impact over time.

Moving forward it will likely be advantageous to leverage components of the process marker model presented by Trochim et al. to evaluate TtP teams [11]. Identifying concrete and readily observable markers for where the project is currently and where it is targeted to be at the end of the 2 year project will allow for more direct attribution of TtP benefits and may allow for comparison to other non-TtP projects. Future work will also include efforts to further streamline the process by more formally calculating readiness assessment scores before team selection. We will also enhance team status reporting by developing a dashboard to better communicate with the TtP goal team about the progress and barriers of each team. Further, the governance structure will be expanded to closely engage other Mayo Clinic departments (e.g. regulatory strategy, provider relations) to facilitate project success. These refinements are anticipated to further solidify TtP support for the complex and rarely linear pathway from discovery to application.

## Conclusions

TtP is effective in advancing biomedical research toward the clinical practice, as evidenced by the high proportion (75%) of teams advancing through clinical trials, commercialisation, or implementation of a clinical service line or diagnostic test. In particular, TtP concepts affecting the success rate include (1) engagement of institutional leaders to remove administrative barriers; (2) rigorous selection of projects with high potential for clinical translation; (3) teamwork across multiple disciplines; and (4) application of structured project management methodology.

TtP is a novel, pragmatic approach that applies formal project management and leadership influence to intentionally translate research findings in an academic medical centre. This approach can be quickly integrated to create an express pathway for prioritised implementation of an organisation’s most important discoveries.

## Data Availability

The datasets used and/or analysed during the current study are available from the corresponding author upon reasonable request.

## Abbreviations

DTA: Discovery–translation–application
ME&C: Management Engineering & Consulting
NIH: National Institutes of Health
PI: Principal investigator
RFA: Request for applications
SWOT: Strengths, Weaknesses, Opportunities or Threats
TRL: Translational readiness level
TtP: Transform the Practice
